# CUL4A expression is associated with tumor stage and prognosis in nasopharyngeal carcinoma

**DOI:** 10.1097/MD.0000000000018036

**Published:** 2019-12-20

**Authors:** Xin Jin, Yong-Chi Ma, Wen-Yan Zhu, Lun Fan

**Affiliations:** Department of Otolaryngology Head and Neck Surgery, The Affiliated Huaian No. 1 People's Hospital of Nanjing Medical University, Huaian, Jinagsu Province, China.

**Keywords:** cullin 4A, nasopharyngeal carcinoma, prognosis, progression

## Abstract

Cullin 4A (CUL4A) is a protein of E3 ubiquitin ligase with many cellular processes. CUL4A could regulate cell cycle, development, apoptosis, and genome instability. This study aimed to analyze the expression of CUL4A in nasopharyngeal carcinoma (NPC) tissues and the associations of CUL4A expression with prognostic significance. A total of 115 NPC patients were collected to assess the protein expression of CUL4A by immunohistochemistry, so as to analyze the relationships between CUL4A expression and clinicopathological and prognostic parameters. All patients were followed-up until death or 5 years. The results showed that high expression of CUL4A was significantly associated with larger primary tumor size (*P* = .026), higher nodal status (*P* = .013), more distant metastasis (*P* = .020), and higher TNM stage (*P* = .005). Kaplan–Meier curves showed that patients with higher CUL4A expression had significantly shorter overall survival (OS) and progression-free survival (PFS) (both *P* < .001). In multivariate Cox analysis, CUL4A is an independent prognostic factor for OS (*P* = .016; hazard ratio [HR] = 2.770, 95% CI: 1.208–6.351) and PFS (*P* = .022; HR = 2.311, 95% CI: 1.126–4.743). In conclusion, high expression of CUL4A was associated with advanced disease status of NPC, and might serve as an independent prognostic factor.

## 1 Introduction

Nasopharyngeal carcinoma (NPC) is a malignant neoplasm within pharyngeal recess of nasopharynx, with special geographic distribution.^[1]^ NPC is mainly reported in southern China, Southeast Asia, North and East Africa, and the Middle East. In China, the estimated incidence rate was 60.6 per 100,000 people, and the estimated mortality rate was 34.1 per 100,000 people.^[2]^ NPC is driven be many related genetic and environmental factors, including Epstein–Barr virus (EBV) infection and genetically controlled biological progresses such as tumor microenvironment.^[3,4]^

Cullin 4A (CUL4A) is a member of conserved cullin proteins family (CUL1, 2, 3, 4A, 4B, 5, and 7), and constitutes an component of E3 ubiquitin ligase.^[5]^ CUL4A acts as a scaffold protein to bind DNA damage binding protein 1 (DDB1), through which constitute ubiquitin ligase E3 complex, so as to mediate the degradation of many substrates, such as DDB2, REDD1, and histone H2A.^[6–8]^ Thus, CUL4A mediates various cellular processes, such as proliferation, differentiation, apoptosis, hematopoiesis, DNA replication, and genomic stability.^[9]^ In recent years, CUL4A has been reported to hydrolyze in a ubiquitin-dependent manner of several tumor suppressor genes, such as p21,^[10]^ p27,^[11]^ and p53,^[12]^ so the role of CUL4A as a potential oncogene has been proposed. In fact, CUL4A gene has been reported to be amplified in various tumors such as hepatocellular carcinoma and breast cancer.^[13,14]^ Furthermore, CUL4A overexpression was found in a variety of cancers, including malignant pleural mesothelioma and pituitary adenomas.^[15,16]^ High CUL4A expression in intrahepatic cholangiocarcinoma,^[17]^ lung cancer,^[18]^ and colorectal cancer^[19]^ correlated with poor prognosis and shorter overall survival (OS). Thus, CUL4A contributes to both tumor initiation and progression, and understanding the mechanisms associated with progression, metastasis, and prognosis might be valuable to serve CUL4A as a prognosis biomarker and a target for drug development.

Currently, the expression of CUL4A has never been evaluated in NPC patients. In the present study, we determined the expression levels of CUL4A in NPC and their paired adjacent nontumor tissues, and investigated the associations of CUL4A expression with key clinicopathological parameters and survival of NPC patients.

## 2 Materials and methods

### 2.1 Study subjects and specimens

A retrospective study was performed in 115 patients with NPC who were treated between July 2010 and June 2013 at the Affiliated Huaian No.1 People's Hospital of Nanjing Medical University. The tumor biopsies were collected before treatment. The NPC was diagnosed and classified according to the guidelines in the 2010 American Joint Committee on Cancer (AJCC, 7th edition). Standard curative radiotherapy was carried out for all NPC patients, and patients with stages II to IV disease also received concurrent chemotherapy (cisplatin). All patients were regularly followed-up for at least 5 years. Overall survival was defined as the start of therapy to death or last follow-up. Progression-free survival (PFS) was defined as the start of therapy to progression (loco-regional relapse or distant metastasis) or last follow-up. Informed consent was obtained from all patients before this study, which was approved by the Ethics Committees of The Affiliated Huaian No.1 People's Hospital of Nanjing Medical University (No. KY-P-2019–029–01; Jiangsu, China).

### 2.2 Immunohistochemistry

Immunohistochemistry (IHC) was performed to determine the protein expression of CUL4A. Briefly, sections (4 μm) were made from paraffin-embedded specimens, followed by antigen retrieval using a microwave oven. Sections were incubated with 0.3% H_2_O_2_ to block endogenous peroxidase, and incubated with 1% bovine serum albumin for blocking nonspecific binding. Then, sections were incubated with anti-CUL4A antibody (1:400, ab34897; Abcam, Cambridge, UK) overnight at 4°C, and then washed with PBS solution and incubated with horseradish peroxidase conjugated secondary antibody at 37°C for 1 hour. In the end, sections were stained with 3,3′-diaminobenzidine (DAB) for 10 minutes and counterstained with 10% hematoxylin. The sections that incubated with the normal goat serum other than primary antibody server as negative controls.

The slides were observed and scored independently by two experienced pathologists who were blinded to the detailed information of patients. The criteria for each score were as follows and the staining intensity was scored in four categories as follows: negative, 0; weak, 1; moderate, 2; and strong, 3. The proportion of positively stained cells was determined as follows: 0–25%, 26–50%, 51–75%, and 76–100%. The final expression level of CUL4A in each sample was determined by multiplying the proportion and the intensity. The cutoff point was median score of 6 and to divide all patients into low expression group (scores of 0–4) and high expression group (scores of 6–12).^[18]^

### 2.3 Statistical analysis

The SPSS 20.0 software was used to perform statistical analysis (SPSS, Inc., Chicago, IL). The χ^2^ test or Fisher's exact test was carried out to analyze the relationship between CUL4A expression and clinicopathological variables of NPC. The Kaplan–Meier analysis was used to compare the survival between NPC patients with high and low CUL4A expression. In addition, the multivariate Cox proportional hazards model was carried out to determine the independent contribution of CUL4A proteins. *P* < .05 was considered as the criteria for statistically significant difference.

## 3 Results

### 3.1 Clinicopathological features of our NPC cohort

Totally 115 NPC patients were analyzed, and there were 68 males and 47 females, with a median age of 49 years (range: 27–71 years). There were 11 cases classified as stage I, 14 cases classified as stage II, 34 cases classified as stage III, and 56 cases classified as stage IV (Table 1). The mean follow-up period was 5 years, and the median duration to tumor-associated mortality and progression was 29 and 26 months, respectively.

**Table 1:**
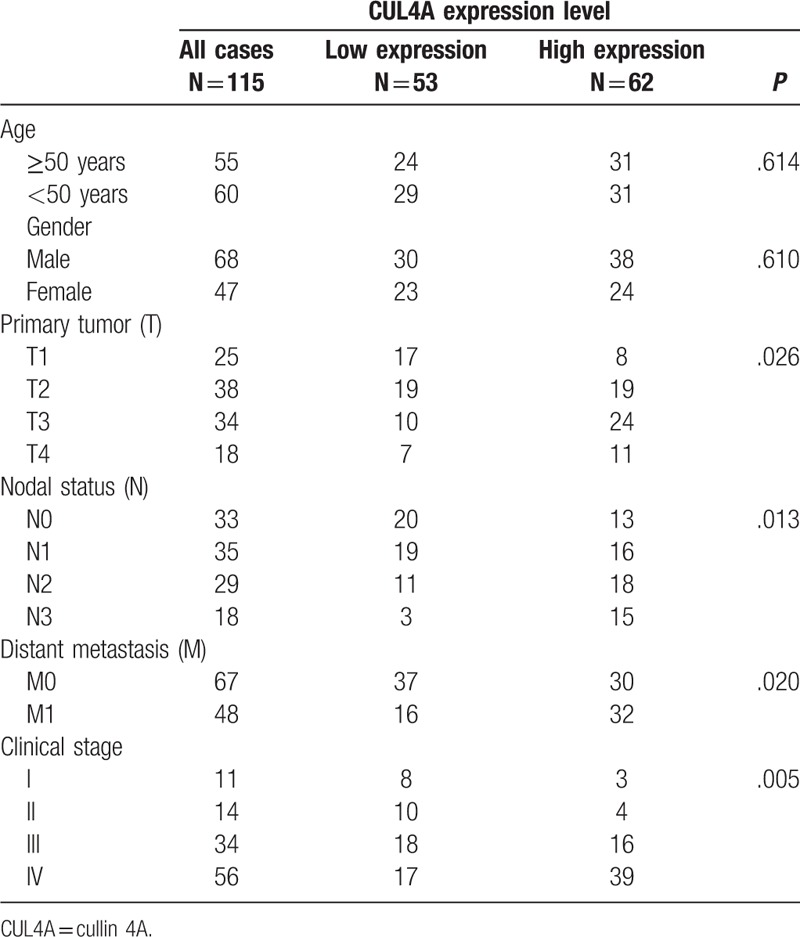
Association between CUL4A expression and clinicopathological variables in nasopharyngeal carcinoma patients.

### 3.2 Expression of CUL4A in NPC as determined by immunohistochemistry

**Figure 1:**
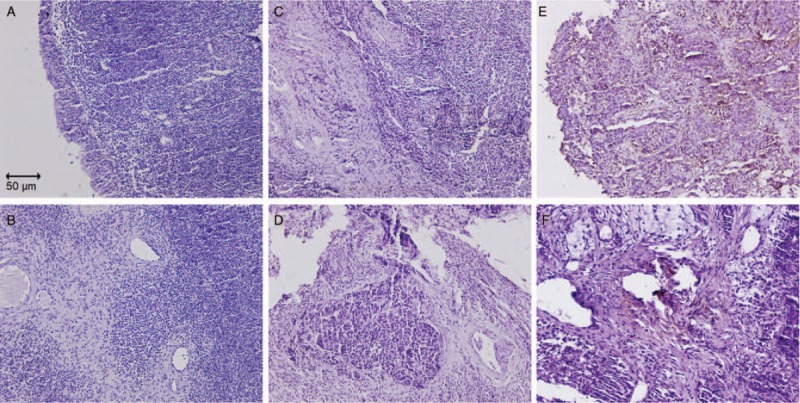
Representative images of CUL4A as detected in 115 paraffin-embedded NPC tissues by immunohistochemistry. In NPC tissues, the tumor cells show weak staining in tumor-adjacent tissues (A and B), low staining in low-stage NPC (C and D), and high staining in high-stage NPC (E and F), respectively. Brown denotes a positive signal. Magnification, A, C, and E: ×200; B, D, and F: ×400.

Immunohistochemistry was performed in tumor tissues and tumor-adjacent tissues from 115 NPC patients. Representative examples of staining showed that there was no CUL4A expression in tumor-adjacent tissues (Fig. 1A and B). However, CUL4A showed low (Fig. 1C and D) and high (Fig. 1E and F) cytoplasmic expression in low-stage (I) and high-stage (IV) of NPC samples. Among 115 cases of NPC, 53 cases had low CUL4A expression and 62 cases had high CUL4A expression, which demonstrated that CUL4A was over expressed in about half (53.9%) NPC.

### 3.3 Correlations of CUL4A expression with clinicopathological parameters of NPC

We investigated the associations between CUL4A expression and clinicopathologic characteristics of NPC patients by χ^2^ test. The results showed that CUL4A expression was significantly correlated with larger tumor size (*P* = .026), and lymph node involvement (*P* = .013), distant metastasis (*P* = .020), and clinical stage (*P* = .005) (Table 1). However, there was no significant correlation of CUL4A expression with age and gender.

### 3.4 Survival analysis

**Figure 2:**
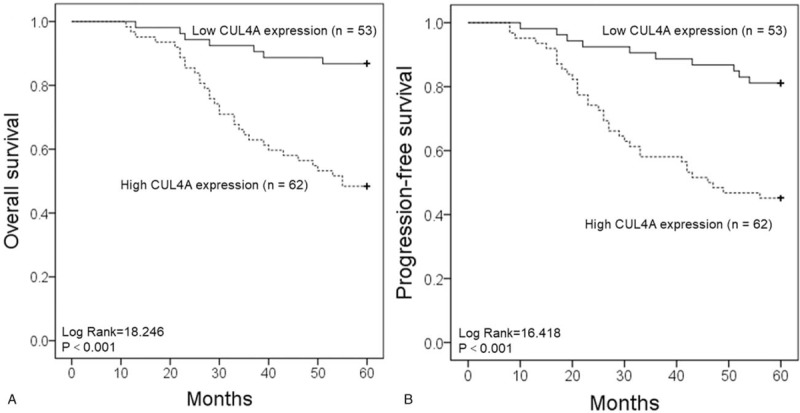
Expression of CUL4A is significantly correlated with survival of patients with NPC. Kaplan–Meier curves estimate the overall survival (A) and progression-free survival (B) rates for 115 NPC patients according to CUL4A expression. *P*-Values were obtained using the log-rank test. NPC = nasopharyngeal carcinoma.

All the 115 NPC patients had intact follow-up for at least 5 years to get intact information for survival analysis. According to the expression levels of CUL4A protein, these patients were divided into two groups: patients with high CUL4A expression and low CUL4A expression. Kaplan–Meier survival analyses showed that NPC patients with high CUL4A protein expression had a significantly lower OS and PFS rates than those with low CUL4A expression (both *P* < .001; Fig. 2A and B). Finally, Cox multivariate analysis determined that CUL4A expression (OS: *P* = .016; PFS: *P* = .022), as well as primary tumor (both *P* < .001), nodal status (OS: *P* = .002; PFS: *P* = .005) and distant metastasis (OS: *P* = .012; PFS: *P* = .017) were independent factors with prognostic value in patients with NPC (Table 2).

**Table 2:**
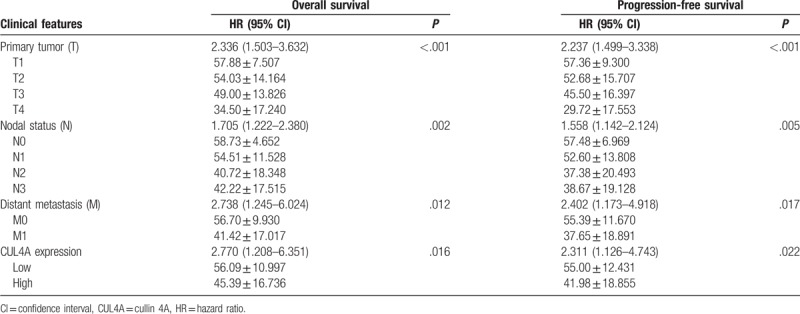
Multivariate Cox regression analyses for overall survival and progression-free survival in nasopharyngeal carcinoma patients.

## 4 Discussion

The present study showed that CUL4A was overexpressed in NPC tissues compared to tumor-adjacent normal tissues. High CUL4A expression was positively correlated with clinicopathological parameters, such as larger primary tumor size, higher incidence of nodal status, distant metastasis, and advanced clinical stage. NPC patients with high CUL4A expression had poor clinical outcome, as evidenced by shorter OS and PFS. Therefore, CUL4A can act as an unfavorable prognostic factor of NPC.

CUL4A is a scaffold protein of ubiquitin ligase E3 complex, and involves the ubiquitination and degradation of tumor suppressor genes, including p21,^[10]^ p27,^[11]^ and p53.^[12]^ Thus, CUL4A has been regarded as an oncogene, but it remains unknown whether CUL4A plays a role in NPC. Our study found that expression of CUL4A was upregulated in tumor tissue of NPC, and is significantly associated with primary tumor size, modal status, distant metastasis, and clinical stage. High CUL4A is a significant independent prognostic factor. These results confirmed CUL4A as a potential oncogene of NPC.

Multivariate Cox proportional hazards regression analysis showed that CUL4A expression could serve as an independent prognostic factor for OS and DFS of NPC. So, the data on CUL4A expression in tumor tissues to help identify patients who may or may not benefit from radiotherapy and chemotherapy, so as to design optimal, individualized treatment for NPC patients.^[20]^ Furthermore, this study also indicates that CUL4A expression may influence the response of radiotherapy and chemotherapy in NPC patients. High CUL4A expression was associated with reduced sensitivity to gemcitabine in lung cancer cells and to cisplatin in colorectal cancer cells.^[21,22]^ Overexpression of CUL4A in normal mammary epithelial cell line MCF10A abrogated the G2/M cell cycle checkpoint and prevented p53 accumulation in response to DNA damage induced by ionizing irradiation, thus contributing tumorigenesis and/or tumor progression.^[23]^ The role of CUL4A in resistance to chemotherapy and radiotherapy can be explained by its regulation on proteolysis of p53. For instance, CUL4A overexpression delayed the accumulation of p53 in response to DNA damage in mouse embryonic fibroblasts, which was dependent on MDM2.^[12]^ Therefore, CUL4A could suppress DNA damage response and promotes cell survival in PC12 cells subjected to hypoxia and reoxygenation.^[24]^ These cells survived from reduced repair proficiency might initiate carcinogenesis and contribute to tumor recurrence after chemotherapy and radiotherapy.^[25]^ The latter hypothesis was also supported by our result that NPC patients with higher CUL4A expression had shorter PFS time.

Chemotherapy is used as induction therapy for NPC patients with advanced stage (II–IV) concurrently with radiotherapy.^[26]^ However, its outcome remains unsatisfactory for recurrent or metastatic subjects, with drug resistance as one main contributing factor. For example, MDR-1 is a membrane protein with multidrug efflux pump function that confers drug resistance for cells exposed to chemotherapeutic drugs. MDR-1 could be detected in tumor cells from 12.6% primary NPC patients and 32.6% recurrent NPC patients.^[27]^ A recent study showed the evidence that overexpression of CUL4A in breast cancer cells increased expressions of MDR-1 mRNA and protein levels and induced drug resistance.^[28]^ These findings indicates an important role of CUL4A in chemotherapeutic drug resistance, and MDR1 might be a downstream protein in a subset of NPC patients.

We also provide evidence that high expression of CUL4A is associated with the larger tumor size, lymph node invasion and distant metastasis. Our results are in accordance with other reports that high CUL4A expression is associated with tumor cell proliferation, lymphatic invasion, and metastasis in hepatocellular carcinomas,^[29]^ lung cancer,^[18]^ and perihilar cholangiocarcinoma.^[30]^ These clinical association between CUL4A expression and clinicopathological features were further confirmed by in vitro study that CUL4A overexpression can promote proliferation and invasion in cultured cells from gastric cancer,^[31]^ osteosarcoma,^[32]^ and ovarian cancer.^[33]^ The mechanisms underlying tumor cell invasion and metastasis are not fully understood, and convincing evidence indicates that epithelial-mesenchymal transition (EMT) has a close relationship with invasion and metastasis of malignant tumor, including NPC.^[34]^ CUL4A overexpression could induce EMT process in normal or malignant human mammary epithelial cells, and increase their neoplastic properties and metastatic capacity.^[35]^ On the contrary, knockdown of CUL4A expression significantly suppressed EMT process, reduced in vitro proliferation, migration and invasion, and in vivo tumor growth of colon cancer cells, which was dependent on ZEB1, an EMT inducer.^[19]^ Furthermore, EMT also promote the resistance to chemotherapy or radiotherapy in NPC.^[36,37]^ However, it remains unclear about the role of CUL4A in invasion, metastasis and EMT of NPC cells and deserves further study.

In conclusion, this study demonstrates that CUL4A expression was upregulated in NPC tissues, and CUL4A high expression showed a significant correlation with tumor size, lymph node involvement, distant metastasis, and clinical stage. Patients with high CUL4A expression had shorted survival time and CUL4A is an independent prognosis factor of NPC. Our findings highlight the possible role of CUL4A in response to chemotherapy and radiotherapy. Further study are needed to explore the molecular alterations regulated by CUL4A in the cellular pathways involved in NPC carcinogenesis, invasion, metastasis, and EMT, which will certainly facilitate the integration of diagnosis and therapy for NPC.

## Author contributions

**Conceptualization:** Wen-Yan Zhu.

**Data curation:** Xin Jin, Yong-Chi Ma.

**Formal analysis:** Lun Fan.

**Investigation:** Xin Jin, Yong-Chi Ma.

**Methodology:** Xin Jin, Yong-Chi Ma.

**Project administration:** Wen-Yan Zhu.

**Resources:** Wen-Yan Zhu.

**Software:** Lun Fan.

**Supervision:** Wen-Yan Zhu.

**Writing – original draft:** Xin Jin.

**Writing – review & editing:** Wen-Yan Zhu.

Wen-Yan Zhu: 0000-0003-4083-052X
